# Risk Factors and Mechanisms Underlying Cross-Shift Decline in Kidney Function in Guatemalan Sugarcane Workers

**DOI:** 10.1097/JOM.0000000000001529

**Published:** 2018-12-20

**Authors:** Cecilia J. Sorensen, Jaime Butler-Dawson, Miranda Dally, Lyndsay Krisher, Benjamin R. Griffin, Richard J. Johnson, Jay Lemery, Claudia Asensio, Liliana Tenney, Lee S. Newman

**Affiliations:** Center for Health, Work & Environment (Dr Sorensen, Dr Butler-Dawson, Ms Dally, Ms Krisher, Ms Tenney, Dr Newman); Department of Emergency Medicine, University of Colorado School of Medicine (Dr Sorensen, Dr Lemery); Colorado Consortium on Climate Change and Human Health (Dr Sorensen, Dr Butler-Dawson, Ms Dally, Ms Krisher, Dr Johnson, Dr Lemery, Ms Tenney, Dr Newman); Department of Environmental and Occupational Health (Dr Butler-Dawson, Ms Dally, Ms Krisher, Ms Tenney, Dr Newman), Colorado School of Public Health; Division of Renal Diseases and Hypertension (Dr Griffin, Dr Johnson); Pantaleon, Guatemala (Mr Asensio); Division of Pulmonary Sciences and Critical Care Medicine, Department of Medicine, School of Medicine (Dr Newman), University of Colorado, Aurora, Colorado.

**Keywords:** agricultural workers, chronic kidney disease, global health, prevention

## Abstract

**Objective:**

Identify early biomarkers and mechanisms of acute kidney injury in workers at risk of developing chronic kidney disease of unknown origin (CKDu).

**Methods:**

We assessed cross-shift changes in kidney function and biomarkers of injury in 105 healthy sugarcane workers. We obtained pre-harvest clinical data as well as daily environmental, clinical, and productivity data for each worker.

**Results:**

The average percent decline in cross-shift estimated glomerular filtration rate (eGFR) was 21.8% (standard deviation [SD] 13.6%). Increasing wet bulb globe temperature (WBGT), high uric acid, decreased urine pH, urinary leukocyte esterase, and serum hyperosmolality were risk factors for decline in kidney function.

**Conclusions:**

Sugarcane workers with normal kidney function experience recurrent subclinical kidney injury, associated with elevations in biomarkers of injury that suggest exposure to high temperatures and extreme physical demands.

## Abstract 2

Supplemental Digital Content is available in the text

Chronic kidney disease of unknown origin (CKDu) is an emerging global pandemic with known hot-spots in agricultural communities of Central America,^1–4^ India,^5,6^ Sri Lanka,^7^ Egypt,^8^ and the United States.^9,10^ In Central America it has become a leading cause of death and hospitalization, particularly among young, male sugarcane workers, but has also been shown to affect women and children in agricultural communities.^11–14^ Between 1990 and 2009, the mortality from CKD increased 7-fold in El Salvador and 3-fold in Nicaragua.^8^ The age-adjusted mortality rates from end stage renal disease (ESRD) in these countries are among the highest in the world.^15^ The Pan American Health Organization (PAHO) estimates that more than 60,000 renal failure deaths (41% in those younger than 60) occurred in Central America between 1997 and 2013.^15,16^ According to projections from the Global Health Observatory, CKD is currently one of the fastest growing causes of global mortality worldwide, and impacts are anticipated to increase over the next decade.^17^

Currently, CKDu is often diagnosed at a very late stage due to a lack of overt early clinical signs and symptoms and a dearth of systematic surveillance.^16^ Recent evidence suggests that some workers at risk for CKDu are experiencing episodes of clinically apparent acute interstitial nephritis with renal biopsies demonstrating tubulointerstitial inflammatory infiltrates with varying degrees of underlying fibrosis.^11,18^ This histological pattern may suggest recurrent injury in individuals with a normal estimated glomerular filtration rate (eGFR). Acute kidney injury is a known risk factor for eventual CKD,^19–21^ however, this progression has not been fully explored in the context of the natural history of CKDu. Similarly, little is known about early asymptomatic changes. A better understanding of early pathophysiologic changes in workers at risk for CKDu will help shed light on the underlying pathogenesis and natural history of CKDu and offers opportunities for early stage prevention. Additionally, such knowledge will equip occupational and environmental medicine (OEM) providers, on the forefront of emerging health issues related to worker populations, with information needed to recognize, respond to, and help mitigate these health threats to workers worldwide.

Chronic exposure to high ambient temperatures, extreme physical exertion, and recurrent dehydration have previously been implicated as contributing factors to the development of CKDu.^12,22–24^ While three cohort studies have examined cross-shift changes in renal physiology among workers in endemic regions, data are still limited on the nephrotoxic impact and mechanism of injury that may result from performing intense physical labor in hot setting.^2,25,26^ Exposure to agrochemicals and heavy metals, silicates, intake of fructose-rich soft drinks, use of non-steroidal anti-inflammatory drug (NSAIDs), tobacco use, and nutritional factors are other suspected causes under broad investigation.^27–30^ These potential risk factors are not mutually exclusive and may operate in concert, simultaneously or sequentially over periods of time, resulting in acute and chronic manifestations of disease.^31,32^ Likely, some factors may exert a stronger impact in certain populations and individuals more than others.

To improve understanding of the earliest signs of renal dysfunction in this occupational setting, we examined endogenous biomarkers of physiologic stress and injury in a healthy cohort of Guatemalan sugarcane workers who, by nature of their occupation, are at risk for CKDu. In distinction from prior studies, our workers were also hydrating themselves with a median of 15 L of water daily, thereby allowing us to look at changes in subjects who were well hydrated. The aims of the current study were to (1) assess changes in renal function across a work shift in a cohort of workers with normal eGFR, (2) assess associations between cross-shift changes in kidney function, biomarkers of injury and demographic, environmental, and clinical characteristics, (3) test several hypothetical physiological pathways of injury depicted in Fig. 1, including: heat stress, dehydration and hypovolemia, muscle breakdown, and tissue hypoperfusion, and (4) to examine whether inflammation is a potential intermediate end-point that is associated with kidney injury. A better understanding of early biomarkers of injury serves to inform interventions aimed at prevention, detection, and diagnosis of subclinical disease. Additionally, an understanding of acute early changes in renal function contributes to a growing understanding of the multifactorial root causes of CKDu.

**FIGURE 1:**
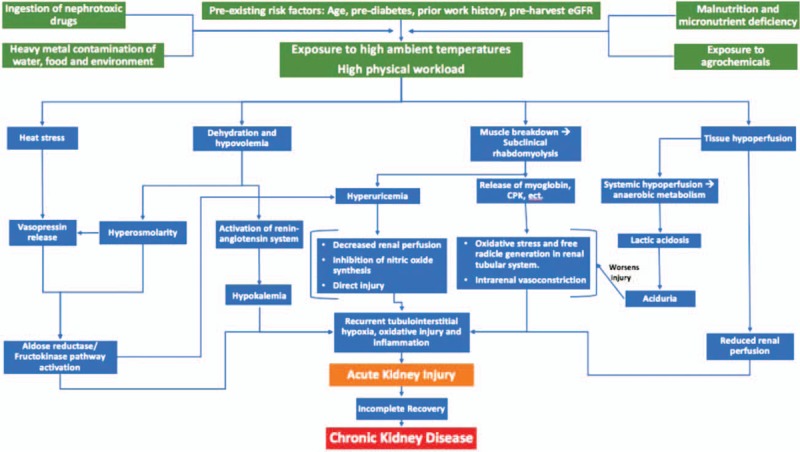
Mechanisms by which recurrent exposure to high ambient temperature and high physical workloads, in synergy with other exposures and risk factors, may lead to CKDu. CKDu, chronic kidney disease of unknown origin.

## MATERIALS AND METHODS

### Setting and Study Design

This study was conducted across the 2016 to 2017 sugarcane harvest in a population of 105 field workers employed by Pantaleon, a sugarcane agribusiness in Guatemala. The sugarcane harvest season runs from November until May each year. A detailed description of the field setting has been described by Butler-Dawson et al.^33^

In this longitudinal study, we prospectively assessed cross-shift changes in eGFR, biomarkers of renal function, and biomarkers of heat stress in 105 workers at three separate time points during the harvest season. Additionally, we collected clinical, environmental, occupational, and non-occupational data for each worker. Prior to each harvest, field workers undergo a fitness-for-work medical screening and health risk assessment survey. The screening evaluation includes clinical data (age, height, weight, blood pressure, heart rate), demographic information (location of home of residence, occupational history, lifestyle behaviors), and evaluation of eGFR via serum creatinine.

Workers were recruited for employment from local communities in the coastal area near the sugarcane fields (approximate altitude 350 m) as well as from highland communities of Guatemala (approximate altitude 1800 m). Workers were hired if they passed the fitness-for-work screening and had an eGFR greater than 60 mL/min/1.73 m^2^. Once hired, workers were assigned to work cohorts of approximately 50 workers each that remained constant for the duration of the season. The University of Colorado Center for Health, Work & Environment (CHWE) and Pantaleon executed a memorandum of understanding in 2016, with the shared goal of assessing and improving the health, safety, and well-being of the workers. Data from the pre-harvest medical screenings were provided to the research team at the CHWE. Ethics review and approval for this study was granted by the Colorado Multiple Institutional Review Board (COMIRB) and in Guatemala by the Comite de Etica, Facultad de Medicina, Universidad Francisco Marroquin-Hospital Universitario Esperanza.

### Study Participants

All participants were over the age of 18 and had passed the pre-employment medical screening. In January 2017, a stratified random sampling method was used to construct a list of 10 work groups. Details are described in Butler-Dawson et al.^31^ In brief, we stratified work groups by job type (production vs cane cutting) and by home residence, local, coastal region residents versus highland region residents (among cane cutting groups only). Eight cane cutting work groups (four local and four highland work groups) were selected out of a total of 46 work groups. Two production groups were selected from 28 work groups that had between 40 and 70 workers. Production workers perform tasks such as planting seeds and field maintenance. Approximately 10 workers from each selected group were randomly chosen for invitation into the study. Of 105 workers who were invited to participate, all 105 were enrolled and participated in at least one of three data collection time points (100%). All study subjects completed and signed a written informed consent if they agreed to participate.

### Productivity and Work Environment

During the harvest season (November to April), cane cutters are typically in the field for 10 hours (cutting cane for 8 hours) in 6-day blocks before taking a rest day. Productivity data for each study participant (tons of cane harvested per day) on the day of study, as well as the prior day, was provided to University of Colorado researchers.

Since 2009, Pantaleon has provided water, rest, and shade (WRS) interventions aligned with US Occupational Safety and Health Administration (OSHA) recommendations.^34^ Cane cutters are instructed to drink 16 L of water including 2.5 L of electrolyte solution (composition per liter: 4.6 g NaCl, 34 g carbohydrates [26 g sucrose], and 2 g KCl) and take three 20-minute work breaks and one 60-minute shaded lunch break during the work shift. Cane cutters are provided with 5 L water jugs and unlimited access to water during the work shift, distributed from centrally located mobile water tanks in the cane field. During the months of February and March, production workers were not included in the WRS intervention. WRS interventions for all production workers began in April. Self-reported data on water and electrolyte solution intake were collected from each worker after each study day.

### Data Collection and Analysis

During study days, evaluations were performed at mobile stations alongside the fields. Urine samples were collected immediately before and after each work shift. Blood was obtained post-shift (venipuncture) and creatinine was measured pre- and post-shift with a handheld point of care (POC) device. Workers were asked to perform their work tasks as they would do on a typical day. Trained bilingual research personnel from the University of Colorado used a structured interviewer-administered survey to evaluate work and health behaviors. Detailed laboratory methods are included as a supplement (Supplemental Digital Content 1).

### Assessment of Kidney Function

Pre- and post-shift blood was collected by finger prick and read instantly in the field using the Nova® Statscan (Stat Sensor Creatinine Meter, Nova Biomedical Corporation, Waltham, MA). In a prior study, we demonstrated that this POC test consistently overestimates creatinine by an average of 22% (95% confidence interval [CI]: 19.8%, 24.7%).^35^ Therefore, an adjustment factor of 0.7775 was applied to all POC creatinine values. eGFR per 1.73 m^2^ of body surface area was calculated using the Chronic Kidney Disease Epidemiology Collaboration formula based on serum creatinine.^36^ The primary outcome for this analysis is cross-shift percent change in eGFR, calculated by comparing pre-shift to post-shift values during the day of study.

### Assessment of Biomarkers

Pre- and post-shift urine samples were collected in sterile cups in the field. On each sample, urine dipstick analysis was performed and specific gravity was measured using a digital refractometer. Samples were subsequently analyzed for creatinine, sodium, potassium, albumin, magnesium, and neutrophil gelatinase-associated lipocalin (NGAL). Urine dipstick results, including pH, leukocyte esterase, and protein, were subsequently analyzed as categorical variables, given the nature of the test.

Post-shift blood samples were collected from each participant by a trained phlebotomist and subsequently analyzed for blood urea nitrogen (BUN), creatinine, uric acid, sodium, potassium, lactic acid, and creatine kinase. Hemoglobin A1c was measured only at the February time point. For biomarkers below the limit of detection, we substituted the limit of detection/√2. To account for urine concentration, we corrected for urinary sodium, magnesium, potassium, and albumin relative to urinary creatinine. Serum osmolality was calculated based on serum electrolytes values.^37^ Normal values for all tests were determined based upon reference laboratory guidelines. See supplement (Supplemental Digital Content 1) for further laboratory methods and collection details.

### Environmental Measures

During each study day, wet bulb globe temperature (WBGT), dry bulb globe temperature (DBGT), and relative humidity were recorded during the work shift using a 3M QUESTemp34 Thermal Environmental Monitor placed 1 m above the ground in direct sun, in the field where the study participants worked. We computed the average and maximum WBGT for each study day. On the last day of the study, the monitor only recorded data for 40 minutes of the work shift so WBGT on this day was not include in the analyses.

### Education and Wellness Incentive

During the informed consent process and at each round of data collection, study subjects received enhanced education that was in addition to the health education modules regularly conducted by company field nurses and doctors. This included a brief presentation and a brochure, based on OSHA's WRS program.^38^ During the study period, workers were incentivized with raffle tickets to maintain adequate hydration (determined by urine specific gravity and change in body weight).^33^

### Statistical Analyses

Baseline characteristics were compared between cane cutters and production workers using chi-square or Fisher Exact tests for categorical characteristics; *t* tests with Satterthwaite corrections for unequal variances were used for continuous variables. Cross-shift percent change in eGFR, was the outcome measure for the univariate and multivariable analyses with all workers combined.

We evaluated the association between potential risk factors and percent change in eGFR across the work shift using linear mixed-effects univariate and multivariable models with random intercepts for individuals. We constructed three multivariable models based on the biomarkers of interest. In model 1, we assessed the associations between pre-shift urinary biomarkers and cross-shift change. In this model, all pre-shift factors that had a *P*-value of <0.1 in the univariate models were included in the final model 1. In model 2, we assessed associations between post-shift biomarkers from the different hypothesized pathways of injury (Fig. 1) and cross-shift change. In this model, we included biomarkers from each of the hypothesized pathways of injury. This model includes average WBGT, serum osmolality, hyperuricemia (serum uric acid more than 7 vs less than or equal to 7), creatine kinase (less than 174, 174 to 870, more than 870), and dipstick pH level (7.0 to 7.5, 6.0 to 6.5, and 5.0). In model 3, we assessed the association between post-shift renal inflammation (dipstick positive leukocyte esterase more than or equal to 70+ leu/μL vs negative/±15) and cross-shift change to assess the hypothesis that recurrent inflammation leads to greater decline in kidney function (percent change in eGFR cross-shift). In all of the multivariable models we controlled for a priori risk factors for decline in kidney function including: age, pre-shift eGFR, hypertension, and HbA1c.^2,12,16,18^ The distribution of cross-shift percent change in eGFR was verified for normality. All analyses were done using SAS version 9.4 (Cary, NC).

## RESULTS

### Participation

Eighty-three cane cutters were invited to participate and all consented. Work attendance varied, thus, 81 workers participated in February, 69 in March, and 71 in April. Sixty-three (76%) cane cutters participated at all three time points. Twelve (15%) participated at two time points and eight (10%) at only one time point. All 22 production workers who were invited to participate consented to the study. Twenty-one participated in February, 22 in March, and 22 in April. Two (9%) production workers participated at only two time points, and 20 (91%) participated at all three time points.

### Pre-Harvest Characteristics

Baseline demographic and clinical characteristics of the 105 workers are shown in Table 1. The mean pre-harvest creatinine for the cohort was 0.90 (SD = 0.15), eGFR = 111.12 (SD = 15.6). In total, 12% of workers started the season with an eGFR less than 90, none below 60. No worker had a HbA1c in the diabetic range (more than 6.5), however, 50% of workers had a HbA1c in the pre-diabetic range (5.7 to 6.4). Evidence of stage 1 hypertension (BP = 140 to 159/90 to 99) was found in 4% of participants. None demonstrated stage 2 hypertension. Differences in pre-harvest clinical variables were not significantly different between the cane cutters and production workers except body mass index.

**TABLE 1:** Pre-Harvest Demographic and Clinical Characteristics of the Study Population

Baseline Characteristics	All Participants *N* = 105	Cane Cutters *N* = 83	Production Workers *N* = 22	*P*-Value
Demographic, mean (SD)				
Age, yrs	30.1 (9.1)	29.05 (8.1)	34.0 (11.3)	<0.05
Home residence, *n* (%)				
Local	63 (60%)	41 (49%)	22 (100%)	<0.01
Highland	42 (40%)	42 (51%)	0 (0%)	
Number of seasons worked	7.1 (5.2)	7.2 (5.0)	6.7 (6.1)	0.21
Clinical, mean (SD)				
Body mass index	23.6 (2.8)	23.2 (2.5)	25.2 (3.1)	<0.01
Pre-harvest serum creatinine, mg/dL	0.9 (0.2)	0.9 (0.2)	0.9 (0.1)	0.5
Pre-harvest eGFR, mL/min/1.73 m^2^	111.1 (15.6)	111.5 (15.8)	109.7 (14.9)	0.6
Pre-harvest eGFR <90, *n* (%)	13 (12.4%)	10 (12.1%)	3 (13.6%)	1.0
Pre-harvest eGFR >90, *n* (%)	92 (87.6%)	73 (87.9%)	19 (86.4%)	
HbA1c^*^	5.66 (0.29)	5.65 (0.28)	5.68 (0.32)	0.75
HbA1c (>6.5) *n*, %^*^	0 (0.00%)	0 (0.00%)	0 (0.00%)	–
HbA1c (5.7–6.4), *n* (%)^*^	53 (50%)	40 (48%)	13 (59%)	0.36
Hypertension^**^	4 (4%)	2 (3%)	2 (9%)	0.22

Median or mean (SD) are shown for continuous variables, number (*n*), and percent (%) for demographics and medical conditions.

^*^HbA1c was measured post-shift in February.

^**^Stage 1 hypertension as defined as a systolic blood pressure from 130-139 or a diastolic blood pressure from 80-89.

### Hydration

As displayed in Table 2, mean self-reported water intake was approximately 13 L daily (median = 15) and mean electrolyte solution intake was approximately five 500-mL bags (2.5 L total fluid) for each worker.

**TABLE 2:** Self-Reported Water and Electrolyte Intake

	Water Intake, L			Electrolyte Solution Intake (500 mL bag)^*^		
	*N*	Mean (SD)	Median (Range)	*N*	Mean (SD)	Median (Range)
February	103	13.8 (6.3)	15 (34.0)	100	5.0 (3.0)	5.0 (12.0)
March	88	13.7 (4.7)	15 (18.0)	88	5.0 (3.4)	5.0 (17.0)
April	92	12.7 (4.4)	15 (18.0)	91	5.4 (3.1)	5.0 (18.0)

^*^Each 500 mL bag of electrolyte solution contains 4 g sodium citrate, 4.6 g sodium chloride, 2 g potassium chloride, 34 g carbohydrates, and 26 g sucrose.

**FIGURE 2:**
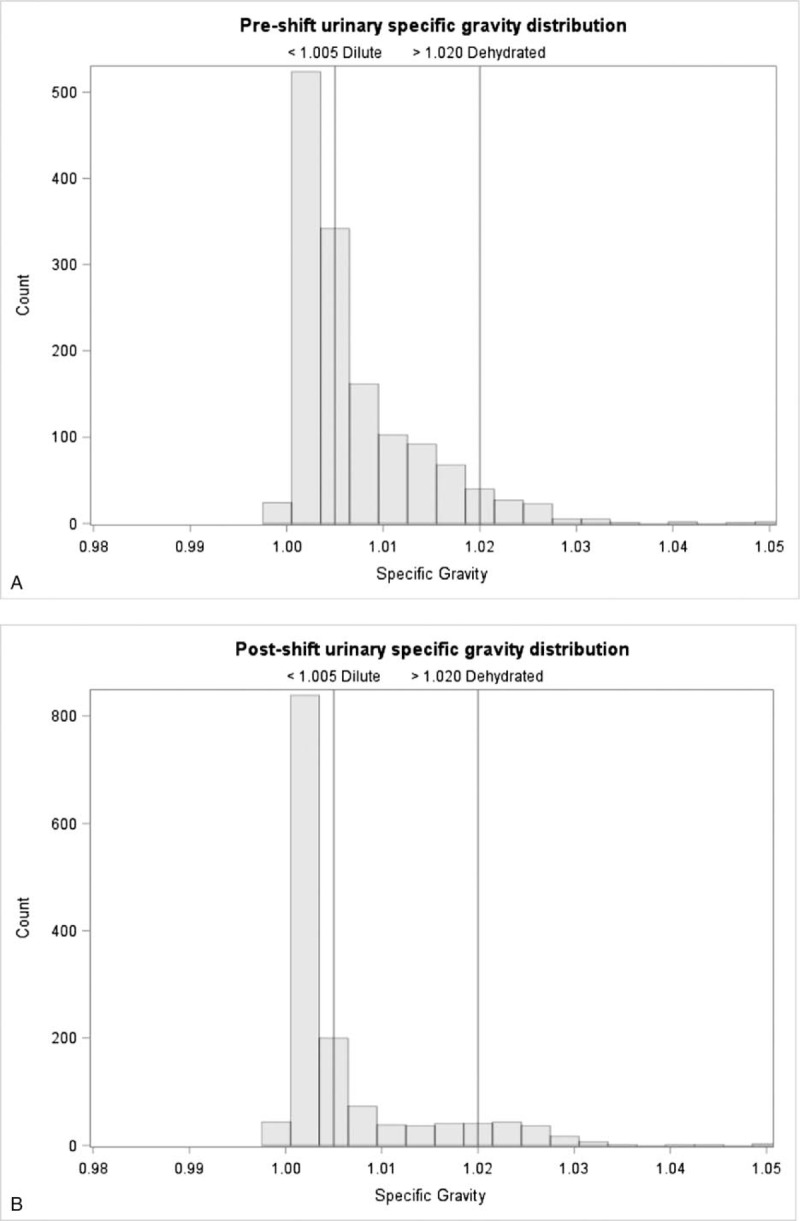
A: Pre-shift urine specific gravity among study participants, averaged across February, March, and April, 2017. B: Post-shift urine specific gravity among study participants, averaged across February, March, and April, 2017.

Overall, workers appeared well-hydrated during this study period. Averaged across the monthly time points, 46% of workers had maximally dilute urine (urine specific gravity less than 1.005) pre-shift, which increased to 67% post-shift. Approximately, 47% started the day with normal urine specific gravity and the proportion decreased to 25% post-shift. Approximately 6% started the day dehydrated (more than 1.02) which increased to 9% post-shift (Fig. 2A, B)

### Cross-Shift Changes in Kidney Function

**FIGURE 3:**
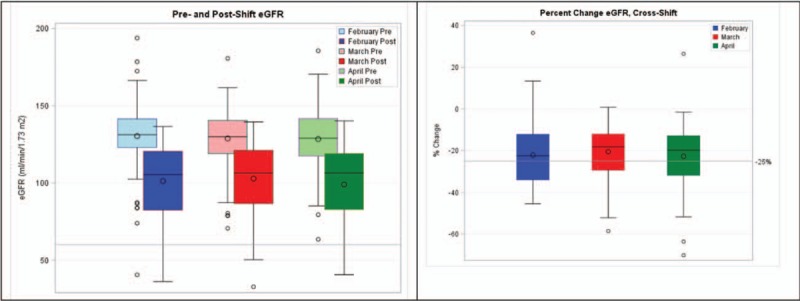
Comparison between pre- and post-shift eGFR during 3 study months (left) and cross-shift percent change in eGFR (right). eGFR, estimated glomerular filtration rate.

Kidney function significantly declined from pre- to post-shift at all time points, *P*-value <0.01 (Fig. 3). Overall, the average percent decline in cross-shift eGFR was 21.8% (SD 13.6%), with 27% to 45% of workers declining by greater than 25% at each monthly time point.

### Heat Exposure and Productivity

The average WBGT temperature was 31.5 °C in February, 30.6 °C in March, and 31.5 °C in April. The average maximum WBGT temperatures were 34.7 °C in February, 34.2 °C in March, and 34.3 °C in April. In February, the average number of tons of cane cut on each study day was 6.2 (SD = 2.3), March 6.6 (SD = 2.5), and April 5.7 (SD = 2.0) (Table 3).

**TABLE 3:** Heat Exposure, Productivity, Behaviors, and Pre- and Post-Shift Urinary Biomarkers for February, March, and April

	February, Mean (SD) or *N* (%)	March, Mean (SD) or *N* (%)	April, Mean (SD) or *N* (%)
Heat exposure			
Average WBGT	31.57 (1.21)	30.67 (1.09)	31.5 (0.7)
Maximum WBGT	34.94 (1.57)	34.25 (1.33)	34.2 (0.7)
Productivity			
Cane harvested on study day (tons/worker)	6.19 (2.25)	6.59 (2.52)	5.7 (2.0)
Can harvested on previous day (tons/worker)	6.31 (2.43)	6.37 (3.46)	5.9 (1.7)
Self-reported behaviors			
Smoked cigarette on study day	5 (5%)	5 (6%)	7 (8%)
NSAID use on study day	12 (12%)	3 (4%)	2 (2%)

Urine Biomarkers	Normal Values	Pre-Shift	Post-Shift	*P*-Value	Pre-Shift	Post-Shift	*P*-Value	Pre-Shift	Post-Shift	*P*-Value
Specific gravity	1.01–1.03	1.008 (0.01)	1.006 (0.01)	**<0.01**	1.009 (0.01)	1.006 (0.01)	**<0.01**	1.007 (0.005)	1.005 (0.006)	**<0.01**
NGAL, ng/mL	^*^	27.1 (51.8)	11.2 (12.6)	**<0.01**	13.8 (17.8)	7.1 (13.1)	**<0.01**	10.6 (11.9)	9.4 (13.4)	**<0.01**
Leukocyte esterase (≥70+ Leu/μL)	0	15 (15%)	4 (4%)	**0.01**	11 (12%)	8 (9%)	**<0.01**	6 (7%)	12 (13%)	**<0.01**
Albumin/creatinine, mg/g	<30	22.3 (68.1)	18.0 (24.1)	0.71	14.0 (28.9)	20.7 (26.4)	**<0.01**	9.1 (13.3)	29.4 (61.4)	**0.71**
pH = 7.0–7.5	5.0–8.0	13 (13%)	14 (14%)	<0.01	16 (18%)	16 (18%)	**<0.01**	10 (11%)	13 (14%)	**<0.01**
pH = 6.0–6.5	5.0–8.0	50 (50%)	50 (50%)		34 (37%)	42 (47%)		48 (52%)	47 (51%)	
pH = 5.0–5.5	5.0–8.0	32 (32%)	37 (37%)		16 (18%)	16 (18%)		34 (37%)	32 (35%)	
Uric acid/creatinine, mg/mg	^*^	0.79 (0.8)	0.49 (0.39)	**<0.01**	0.58 (0.46)	0.37 (0.15)	**<.001**	0.52 (0.15)	0.4 (0.15)	**<0.01**
Urine uric acid, mg/dL	^*^	15.7 (12.3)	10.9 (11.8)	**<0.01**	17.3 (10.0)	11.3 (11.7)	**<.001**	24.4 (19.3)	13.0 (15.0)	**<0.01**
Sodium/creatinine, mmol/g	^*^	236.7 (242.2)	156.5 (216.5)	**<0.01**	121.9 (101.3)	111.4 (95.3)	0.1	125.1 (197.7)	105.5 (163.4)	0.17
Magnesium/creatinine, mg/mg	^*^	0.14 (0.14)	0.11 (0.06)	**<0.001**	0.14 (0.11)	0.11 (0.06)	**0.03**	0.20 (0.1)	0.10 (0.1)	**<0.001**
Proteinuria (≥30 mg/dL)	0	0 (0%)	2 (2%)	–	1 (1%)	1 (1%)	–	0 (0%)	1 (1%)	–

Bold values are statistically significant.

^*^Normal values not established.

### Cross-Shift Changes in Urine Biomarkers

Table 3 displays the average pre-shift and post-shift urine and serum biomarkers for all workers at each time point. Urine specific gravity deceased significantly across the work shift. Average NGAL levels ranged from 10 to 27 ng/mL pre-shift to 7 to 11 ng/mL post-shift and significantly decreased across the work shift. Urinary leukocyte esterase was a common finding both pre- and post-shift. Average albumin to creatinine ratio (ACR) ranged from 9 to 22 μg/mg pre-shift to 18 to 30 μg/mg post-shift and only increased significantly across the work shift in March. Aciduria (defined as a dipstick pH of 5.0) was a common finding both pre- and post-shift. Urinary sodium and magnesium decreased significantly across the work shift.

### Post-Shift Serum Biomarkers

Creatine kinase as well as lactic acid levels were elevated at all time points (Table 4). Average uric acid levels were on the high end of normal (6.2 to 6.4), with 18% to 24% of workers demonstrating hyperuricemia (more than 7.0 mg/dL). Average glucose levels were normal. BUN ranged from 12 to 15 mg/dL. Hypo-osmolality was common, with 31% to 51% of workers demonstrating a serum osmolality less than 280 mmol/kg. Very few workers demonstrated hyperosmolality (osmolality more than 295 mmol/kg). Average serum potassium was low, ranging from 3.4 to 3.5 mmol/L (normal = 3.5 to 5.0). Average serum sodium was on the low end of normal, ranging from 134 to 136 (normal = 135 to 145).

**TABLE 4:** Post-Shift Biomarkers of Injury From February, March, and April, 2017

		February	March	April
Post-Shift Serum Biomarkers	Normal Values	Mean (SD) or *N* (%)	Mean (SD) or *N* (%)	Mean (SD) or *N* (%)
Creatine kinase	174 (IU/L)	436.7 (241.3)	339.9 (239.6)	425.0 (370.0)
Lactate	0.5–1 (mmol/L)	3.8 (1.4)	3.34 (1.03)	3.9 (0.9)
Uric acid	3.4–7.0 (mg/dL)	6.2 (2.4)	6.16 (2.19)	6.4 (1.3)
Uric acid >7.0		18 (18%)	18 (23%)	24 (28%)
Glucose	70–100 (mg/dL)	81.8 (13.7)	85.4 (13.5)	88.4 (15.6)
Blood urea nitrogen (BUN)	7–20 (mg/dL)	14.8 (5.7)	12.9 (4.3)	13.4 (5.2)
Osmolality <280	280–295 (mmol/kg)	51 (51%)	34 (43%)	31 (36%)
Osmolality >295	280–295 (mmol/kg)	1 (1%)	1 (1%)	2 (2%)
Potassium	3.5–5.0 (mmol/L)	3.5 (0.46)	3.4 (0.40)	3.5 (0.40)
Sodium	135–145 (mmol/L)	134.39 (3.77)	135.35 (3.29)	136.00 (3.50)
ΔIntracellular volume	(L)^*^	0.95 (0.66)	0.78 (0.57)	0.66 (0.58)
ΔExtracellular volume	(L)^*^	−1.00 (1.79)	−0.68 (1.40)	−0.24 (2.33)

^*^Normal values not established.

### Factors Associated With Cross-Shift Decline in eGFR

#### Baseline Demographics and Clinical Characteristics

As shown in Table 5, we observed that for each year increase in age, the cross-shift eGFR declined an average of 0.3% (*P* < 0.01). Similarly, cross-shift eGFR declined with each number of sugarcane harvests worked (*P* < 0.01). Those with a higher pre-season baseline eGFR suffered less cross-shift decline in eGFR (*P* < 0.0001). Although none of the study participants met the laboratory definition of diabetes (based on HbA1c at the start of the study), for each 1% increase in HbA1c, there was an average 7.8% decline in cross-shift eGFR (*P* < 0.01). The presence of hypertension was not significantly associated with decline. Pre-shift eGFR was not associated with cross-shift declines. Home residence and body mass index were not significantly associated with cross-shift declines.

**TABLE 5:** Univariate Analysis Testing Associations Between Risk Factors and Percent Change in eGFR Across the Work Shift

Effect	Estimate	Standard Error	DF	*t* Value	*P*-Value
Demographics and baseline health					
Age	**−0.3**	**0.1**	**103.0**	**−2.5**	**<0.01**
Harvests worked (*n* = 96)	**−0.5**	**0.2**	**94.0**	**−2.6**	**<0.01**
Highland	−1.4	2.0	81.0	−0.7	0.51
Body mass index, kg/m	−0.3	0.4	95.0	−0.9	0.40
HgA1c	**−7.8**	**3.4**	**103.0**	**−2.3**	**0.02**
Hypertension (stage 1)	−0.2	5.5	95.0	0.0	0.96
Pre-harvest eGFR	**0.2**	**0.1**	**102.0**	**3.7**	**<0.01**
Pre-Shift eGFR	0.0	0.0	175.0	0.6	0.54
NSAID use	1.2	3.0	100	0.4	0.69
Smoking	−2.7	3.5	158	−0.8	0.44
Heat exposure^*^					
Average WBGT	−0.6	0.7	165	−0.8	0.44
Maximum WBGT	−0.2	0.6	165	−0.4	0.73
Physical work load					
Cane harvested	0.0	0.4	132.0	−0.1	0.90
Cane harvested (prior day)	−0.5	0.4	62.0	−1.2	0.22
Pre-shift urine biomarkers					
Specific gravity	**−2.6**	**1.2**	**174.0**	**−2.3**	**<0.01**
NGAL	**−0.2**	**0.1**	**173.0**	**−2.9**	**<0.01**
Leukocyte esterase, ≥70+/μL	−2.9	2.7	84.0	−1.1	0.29
Albumin–creatinine ratio	0.0	0.0	168.0	0.5	0.60
pH = 6.0–6.5 (ref: 7.0–7.5)	**−3.3**	**1.7**	**78**	**−2.0**	**0.05**
pH = 5.0 (ref: 7.0–7.5)	−3.2	2.5	78	−1.3	0.20
Uric acid	**−0.1**	**0.1**	**168.0**	**−2.4**	**0.02**
Sodium/creatinine	**0.0**	**0.0**	**168.0**	**2.2**	**0.02**
Magnesium/creatinine	**26.5**	**6.1**	**168.0**	**4.4**	**<0.01**
Proteinuria >1+	4.7	12.8	173.0	0.4	0.71
Post-shift urine biomarkers					
Specific gravity	**−3.3**	**1.2**	**175.0**	**−2.7**	**<0.01**
NGAL	**−0.2**	**0.1**	**171.0**	**−2.8**	**<0.01**
Leukocyte esterase, ≥70+/μL	**−7.8**	**2.9**	**94.0**	**−2.7**	**<0.01**
Albumin–creatinine ratio	0.0	0.0	161.0	−0.9	0.36
pH = 6.0–6.5 (ref: 7.0–7.5)	−1.8	1.7	68	−1.0	0.30
pH = 5.0 (ref: 7.0–7.5)	**−9.9**	**2.1**	**68**	**−4.0**	**<0.01**
Uric acid	−0.1	0.1	162.0	−1.8	0.08
Sodium/creatinine	0.0	0.0	161.0	1.8	0.07
Magnesium/creatinine	23.6	14.7	162	1.6	0.11
Proteinuria >1+	2.7	6.8	174	0.4	0.70
Post-shift serum biomarkers					
Creatine kinase, continuous	0.0	0.0	157.0	1.2	0.25
Creatine kinase, 174–870 (ref: ≤174)	4.	2.6	25	1.6	0.23
Creatine kinase, ≥870 (ref: ≤174)	5.5	4.3	25	1.3	0.21
Lactate	0.0	0.7	158.0	−0.1	0.95
Uric acid	**−1.0**	**0.4**	**157.0**	**−2.5**	**<0.01**
Hyperuricemia (>7.0)	**−9.3**	**2.0**	**157.0**	**−4.6**	**<0.01**
Glucose	0.0	0.1	100.0	−0.3	0.76
Blood urea nitrogen	**−0.9**	**0.2**	**157.0**	**−5.7**	**<0.01**
Serum osmolality (cont.)	**−0.3**	**0.1**	**157.0**	**−2.6**	**0.01**
Serum osmolality <280	3.9	1.7	40.0	2.3	0.03
Serum osmolality >295	**−9.1**	**6.5**	**40.0**	**−1.4**	**0.17**
Potassium	2.0	2.1	157.0	1.0	0.34
Sodium	−0.3	0.2	158.0	−1.1	0.28
ΔIntracellular volume	1.9	1.4	158.0	1.3	0.18

Bold values are statistically significant. eGFR, estimated glomerular filtration rate; NGAL, neutrophil gelatinase-associated lipocalin; NSAID, non-steroidal anti-inflammatory drug.

^*^Last study day removed from analysis because WBGT meter only ran for 40 minutes during the work shift.

#### Heat Exposure and Productivity

In the univariate model, neither average or maximum WBGT was significantly associated with cross-shift declines in eGFR. The quantity of cane harvested, either on the day of study or the day prior, was also not significantly associated with cross-shift changes in eGFR.

#### Urine Biomarkers

Increasing pre- and post-shift specific gravity were significantly associated with a cross-shift decline in eGFR (*P* < 0.01). Post-shift aciduria (pH 5.0) was significantly associated with cross-shift decline. For each unit increase in pre- and post-shift NGAL, cross-shift eGFR declined on average 0.2% (*P* < 0.01). Higher urine uric acid levels, both pre- and post-shift, were associated with decline (*P* < 0.1). Post-shift urine leukocyte esterase, more than or equal to 70+ Leu/μL or greater, were associated with an 8% decline in eGFR (*P* < 0.01). Albumin/creatinine ratios and proteinuria (>1+) were not significantly associated.

#### Post-Shift Serum Biomarkers

Uric acid was significantly associated with cross-shift decline in eGFR, and those with hyperuricemia (more than 7.0 mg/dL) experienced an average 9% decline in cross-shift eGFR. BUN was significantly associated with cross-shift decline in eGFR, with every 1 mg/dL increase associated with an approximately 1% decline. Increasing serum osmolality was associated with a 0.3% decline. Serum hypo-osmolality was associated with a 4% increase in eGFR whereas hyperosmolality was not significant although very few workers had hyperosmolality. Creatine kinase, lactate, potassium, and sodium were not significantly associated with cross-shift decline in eGFR.

### Multivariable Analysis of Factors Associated With Cross-Shift Decline in eGFR

Pre-shift biomarkers associated with percent change in eGFR across the work shift are presented in Table 6. Increasing HbA1c and age were significantly associated with cross-shift decline in eGFR in the pre-shift biomarkers model. Increasing urinary magnesium/creatinine was found to be protective and was associated with improvement in kidney function across the work shift.

**TABLE 6:** Multivariable Analysis of Pre-Shift Biomarkers Associated With Percent Change in Cross-Shift eGFR

Effect	Estimate	*P*-Value
Intercept	12.60	0.93
Age (per 1-yr increase)	**−0.26**	**0.04**
HgA1c (per 1% increase)	**−7.35**	**0.04**
Pre-shift eGFR (per 1 ml/min/1.73 m^2^ increase)	−0.04	0.51
Hypertension (ref: no)	1.06	0.84
Pre-shift specific gravity (per 0.1 increase)	0.17	0.90
Pre-shift NGAL	−0.15	0.09
Pre-shift pH = 6.0–6.5 (ref: 7.0–7.5)	−2.64	0.13
Pre-shift pH = 5.0 (ref: 7.0–7.5)	−0.90	0.73
Pre-shift urinary uric acid/creatinine	2.55	0.18
Pre-shift urinary magnesium/creatinine	**17.67**	**0.03**
Pre-shift urinary sodium/creatinine	−0.001	0.81

Bold values are statistically significant.

Next, we created a multivariable model which assessed the contribution of different pathways of injury to decline in cross-shift kidney function (Table 7). In the model, the biomarkers of physiologic pathways of injury included: average WBGT (heat stress), hyperosmolality (dehydration and hypovolemia), hyperuricemia, creatine kinase, and aciduria (muscle breakdown, subclinical rhabdomyolysis, and tissue hypoperfusion). We observed that increasing average WBGT was significantly associated with cross-shift decline in eGFR while controlling for other pathways of injury. Additionally, increasing age, increasing pre-shift eGFR, hyperuricemia, and aciduria were associated with a greater decline in eGFR. Notably, hyperuricemia was the strongest contributor to eGFR decline.

**TABLE 7:** Multivariable Analysis of Post-Shift Biomarkers Associated With Percent Change in Cross-Shift eGFR

Effect	Estimate	*P*-Value
Intercept	150.25	<0.01
Age (per 1-yr increase)	**−0.4177**	**<0.01**
HgA1c (per 1% increase)	−4.4031	0.23
Pre-shift eGFR (per 1 mL/min/1.73 m^2^ increase)	**−0.1738**	**<0.01**
Hypertension (ref: no)	6.5354	0.22
Average WBGT (per 1 °C increase)	**−1.6231**	**0.03**
Osmolality (per 1-unit increase)	−0.2124	0.08
Hyperuricemia (ref: no)	**−8.8987**	**<0.01**
Creatine kinase, 174–870 (ref: ≤174)	1.6358	0.53
Creatine kinase, ≥870 (ref: ≤174)	1.4272	0.74
pH = 6.0–6.5 (ref: 7.0–7.5)	−1.6484	0.35
pH = 5.0 (ref: 7.0–7.5)	**−6.0508**	**0.03**

Bold values are statistically significant. WBGT, wet bulb globe temperature.

In the model which examined the hypothesis that inflammation is associated with daily sub-clinical kidney injury, we observed that post-shift urinary leukocyte esterase was associated with an average decline of 10% across the work shift. In addition, increasing age, HgA1c, and pre-shift eGFR were significant risk factors for decline (Table 8).

**TABLE 8:** Multivariable Analysis of Post-Shift Inflammation Associated With Percent Change in Cross-Shift eGFR

Effect	Estimate	*P*-Value
Intercept	50.55	0.04
Age (per 1-yr increase)	**−0.35**	**<0.01**
HgA1c (per 1% increase)	**−8.24**	**0.02**
Pre-shift eGFR (per 1 mL/min/1.73 m^2^ increase)	**−0.11**	**0.03**
Hypertension (ref: no)	0.54	0.92
Post-shift leukocyte esterase (≥70+ /μL)	**−9.95**	**<0.01**

Bold values are statistically significant. eGFR, estimated glomerular filtration rate.

### Post-hoc Analysis

Creatine kinase may hypothetically produce cross-shift increases in measured serum creatinine. However, in this study, no associations between creatine kinase categories (less than or equal to 174, 174 to 870, more than 870) and percent change in eGFR, nor post-shift creatinine, were found at any time point using Wilcoxon rank test (*P* = 0.90).

## DISCUSSION

In this study of 105 sugarcane workers who started the harvest season with normal eGFR, we saw a high prevalence of acute cross-shift declines in eGFR. We observed that most workers were well hydrated throughout the work shift, yet still experienced significant decline in renal function. The combined evidence relating kidney function to WBGT and other biomarkers of injury leads us to conclude that heat stress is a contributory risk factor for acute kidney injury in this population. Our data also suggest that the daily acute changes in kidney function are the consequence of an interstitial inflammatory process, which is consistent with the current understanding of CKDu.^10^

Additionally, we found surprisingly high rates of pre-diabetes based on HbA1c in this work group which were associated with a significant risk of cross-shift decline in eGFR. One potential reason for this finding is that hyperuricemia has been epidemiologically and experimentally associated with the development of prediabetes and diabetes.^39^ It is also possible that laboratory measurement of HgA1c is being affected by anemia, which is common,^21,40,41^ although not measured in this study. Other risk factors for greater decline across the work shift included age and pre-shift eGFR.

The finding of cross-shift decline in renal function is consistent with other studies of workers exposed to comparable occupational conditions in El Salvador,^2,12^ Nicaragua,^25,42^ the US,^43^ and Brazil.^26^ However, our cohort is noteworthy in that all workers started the season with normal eGFR, were overall well hydrated and despite which, they manifested cross-shift renal dysfunction.

### Role of Heat Stress in Early Kidney Injury

In multivariate analysis, we found WBGT to be a significant contributor to cross-shift decline in kidney function (Table 6). High ambient temperatures coupled with a high physical workload place profound physiologic demands on the body. Previous studies in Central America have suggested a link between these factors and the development of CKDu^2,22,25,26,42,44–46^ and prior studies have found WBGT and heat strain to be significantly associated with cross-shift declines in eGFR.^2,43^ Cutting sugarcane is strenuous work and the average worker in this study harvested over 6 tons of sugar cane per day under an average WBGT of 31.24 (standard deviation [SD] 1.09).^24^ The OSHA heat exposure threshold is 30 °C, at which point it is recommended that workers spend 25% time working and 75% of time resting each hour, whereas these workers spent 10 hours in the field harvesting with 2 hours of rest time (75% time working and 25% time resting).^47^ While our study supports the conclusion that heat exposure contributes to risk of acute kidney injury, we found no relationship between acute renal dysfunction and rhabdomyolysis, suggesting the need to consider other mechanisms of injury.

In addition to contributing to dehydration, hypovolemia, and tissue hypoperfusion, heat exposure independently activates other pathways of injury (Fig. 1). Animal models suggest that heat exposure results in direct vasopressin release, which activates the aldose-reductase/fructokinase pathway, resulting in direct kidney injury from elevated fructose levels and secondary injury from elevated uric acid.^48^

### Role of Uric Acid in Early Kidney Injury

In this study, there was a significant association between hyperuricemia, uricosuria, and decline in cross-shift eGFR. Hyperuricemia was found in approximately 25% of workers, similar to the incidence reported in a cohort of equivalently young and otherwise healthy workers who met clinical criteria for acute CKDu.^11^ Notably, in the multivariable model, it was the single strongest contributor to cross-shift decline in kidney function. Other studies have reported higher rates of hyperuricemia (75% [49] and 91%^2^), however, in those studies the workers did not have a normal baseline eGFR and were potentially identified at later stages of disease.^49^ We suspect that hyperuricemia may be an important early manifestation of kidney injury and plays a role in the pathogenesis of disease.

Mechanistically, heat stress and hypoperfusion result in the release of uric acid from the breakdown of damaged cells and heat exposure results in uric acid generation through the aldose-reductase/fructokinase pathway.^48,50^ Uric acid mediates kidney injury in a number of ways. Systemic hyperuricemia alters autoregulation of intra-renal perfusion pressure, causing afferent arteriolar vasoconstriction, and resulting in an elevation of glomerular pressure. Ultimately, this results in a reduction in renal blood flow, which worsens hypoperfusion and may result in inflammatory impacts and oxidative damage.^51^ Uric acid also suppresses the synthesis of nitric oxide, causing vasoconstriction in the renal tubules, resulting in a hypoxic state, an inflammatory response, and ultimately fibrosis.^52^ Additionally, uricosuria independently induces inflammation resulting in tubular injury and fibrosis, which is worsened under acidic urinary conditions.^53,54^

### Role of Subclinical Rhabdomyolysis in Early Kidney Injury

Chronic clinical and subclinical rhabdomyolysis were common in this cohort, as has been reported elsewhere. Wegman et al^12^ showed that increasing creatine kinase levels correlated with larger cross-shift declines in eGFR. Using a different approach, Paula Santos et al^26^ found significant increases in creatine kinase across the work shift associated with both acute kidney injury (defined per KDIGO criteria^55^) as well as non-acute kidney injury. Using an approach similar to Wegman, we found no relationship between creatine kinase elevations and cross-shift declines in eGFR. Serum creatine kinase begins to rise within 2 to 12 hours following the onset of muscle injury and reaches its maximum within 24 to 72 hours and a decline is usually seen within 3 to 5 days of cessation of injury.^56,57^ Although subclinical rhabdomyolysis was commonly found in our study cohort, data from Clarkson et al^58^ suggest that exercise-induced rhabdomyolysis should not be expected to cause increases in serum creatinine. In fact, given the extent of urinary acidification in our cohort, the lack of relationship between creatine kinase levels and acute nephrotoxicity furthers the case against subclinical rhabdomyolysis as a cause of the acute kidney injury that we observed.^55^

### Role of Tissue Hypoperfusion in Early Kidney Injury

Our findings suggest that tissue hypoperfusion and resulting aciduria may compound the nephrotoxic effects of uric acid and possibly other nephrotoxins. Tissue hypoperfusion results in lactic acid generation from anaerobic cellular metabolism. In this study, poor tissue perfusion was demonstrated by significantly elevated BUN levels that were associated with declining cross-shift eGFR. Poor perfusion results in lactic acidosis and subsequent aciduria (pH < 7.0), which was significantly associated with cross-shift declines in eGFR (Table 5). Additionally, renal hypoperfusion can result in direct injury.^59^

Among this study cohort, post-shift urine alkalinity was significantly associated with an increase in cross-shift eGFR (protective) and elevated post-shift urine specific gravity was associated with a decrease in cross-shift eGFR.

### Role of Dehydration and Hypovolemia in Early Kidney Injury

In this study, indicators of hypovolemia were associated with cross-shift declines in eGFR in the univariate analysis, including increasing post-shift urine specific gravity, BUN, and calculated serum osmolality. Prior studies have suggested that recurrent dehydration is a risk factor for the development of CKDu.^12,32^ Recurrent volume depletion causes a pre-renal pattern of kidney injury which has been shown experimentally to result in renal tubular injury and fibrosis.^59^ Mechanistically, hypovolemia results in increased serum osmolality which stimulates the aldose-reductase pathway in the kidney.^32^ Subsequent fructokinase activity generates oxidants that cause local tubular injury and simultaneously results in the formation of uric acid.^48^ Volume depletion also may cause renal injury by decreasing renal perfusion.^59^

The observed relationship between cross-shift decline in eGFR and serum osmolality and other markers of dehydration may be partially mediated by vasopressin. Recurrent dehydration and resulting renal injury, has been shown to be mediated by vasopressin, an endogenous hormone that is released in response to hyperosmolality and hypovolemia.^48^ Hypovolemia also activates the renin-angiotensin-aldosterone system (RAAS). Aldosterone decreases renal absorption of potassium, which may explain the high prevalence of hypokalemia in this study and others.^1^ Hypokalemia can in-turn independently reduce intrarenal perfusion pressure which can elicit renal tubular injury.^60,61^ Aldosterone release also results in bicarbonate reabsorption in the distal renal tubule which can worsen aciduria.^2^

### Relationship Between Acute, Early Injury, and Chronic Kidney Disease

The proposed pathways described in Fig. 1 converge to suggest a shared mechanism of injury: renal hypoxia, oxidative damage, and resulting inflammation. Supporting this hypothesis, we found evidence that inflammation (leukocyturia) is a significant marker of cross-shift decline in kidney function (Table 7).

Recent evidence suggests that acute kidney injury is highly associated with eventual progression to CKDu. In a recent study by Fischer et al,^21^ 8.4% of patients from CKDu endemic regions who presented to clinical attention with AKI progressed to CKDu within a median of 8 months. Importantly, 98% of patients presented in the acute period with leukocyturia and leukocytosis, suggesting an underlying inflammatory process of injury. Although workers in our study did not require clinical attention, we observed significant leukocyturia at all time points, suggesting that a similar injury pattern is occurring in workers with subclinical injury and that this injury pattern is occurring repeatedly throughout the harvest season. We speculate that workers who experience renal injury 1 day have insufficient time to recover kidney function before returning to work, resulting in compounding injury via the mechanisms described above, eventually leading to CKDu.

### Biomarkers Predictive of Kidney Injury

This study identified an association between several physiologic biomarkers of tubular kidney injury and cross-shift decline in kidney function. In the univariate analysis, we found that both pre- and post-shift urinary NGAL levels significantly correlated with a decrease in cross-shift eGFR. Similarly, in a recent, prospective, community based, longitudinal study in Nicaragua, NGAL was found to be higher in people at high risk of decline in kidney function.^62^ In another longitudinal cohort study among sugarcane workers in Nicaragua, NGAL was found to increase during the harvest relative to pre-season evaluation,^42^ suggesting to the authors that the injury was due to occupational exposures. Urinary NGAL is a tubular epithelial cell protein that is upregulated and released in response to injury.^42^ NGAL's proposed function is as an endogenous bacteriostatic protein that scavenges bacterial siderophores (small molecules which bind iron). By binding iron, NGAL could have a bacteriostatic and antioxidant effect.^63^ Thus, induction of urinary NGAL under harmful conditions is a compensatory response to ameliorate oxidative stress-mediated damage.

From a practical, cost-effective perspective, it is noteworthy that several biomarkers that can be measured by dipstick were elevated in workers who experienced cross-shift declines in eGFR. Thus, dipstick analysis may prove to be a useful on-site screening tool in worker health programs to identify at risk workers and prevent injury. For example, pre-shift and post-shift urinary leukocytes were significantly associated with a decline in eGFR, suggesting an acute, recurrent inflammatory process. There is evidence that CKDu may manifest in an acute fashion at which time the pathologic picture is consistent with acute tubule-interstitial nephritis with accompanying systemic inflammation, including fever, elevations in C-reactive protein, leucocytosis, and culture negative leukocyturia.^11^ Therefore, workers with leukocyturia may be experiencing some degree of acute interstitial nephritis, consistent with acute CKDu.

OEM providers are uniquely positioned to monitor workers for early signs of CKDu, educate workers and employers, and evaluate and treat acute and chronic kidney injury in worker populations. With increasing ambient temperatures due to climate change, the OEM provider can play a pivotal role in recommending heat warning systems, hydration interventions, and medical surveillance programs, using these newly elucidated biomarkers of injury, to limit the burden of disease.^64^

## LIMITATIONS

The study has several limitations. It would have been advantageous to have biomarker data for sequential days to evaluate if injury patterns are cumulative across a work week. Additionally, it would have been useful to have examined serum markers of injury pre-shift to be able to compare cross-shift changes, however, twice per day venipuncture exceeded workers’ comfort. While we recognize that eGFR may not be the ideal indicator of renal function, since serum creatinine is not in equilibrium across the work shift, given the current limited understanding of early markers of kidney injury, such as NGAL, and the use of eGFR in other worker cohort studies evaluating workers that included individuals with normal eGFR,^12,25,26^ we felt it was the best option for the current study design.

## CONCLUSION

In conclusion, the present study demonstrates repetitive subclinical kidney injury among well hydrated sugarcane workers. We found elevations in biomarkers that support several hypothetical pathways through which exposure to high temperatures may contribute to the pathogenesis of kidney damage. However, these pathways of injury almost certainly synergize with other environmental exposures. Future research longitudinal research should test this hypothesis. In the interim, efforts should be made to identify, and mitigate, the risk of recurrent acute kidney injury.

## Supplementary Material

Supplemental Digital Content
