# Supplementary material for: Risk Factors and Mechanisms Underlying Cross-Shift Decline in Kidney Function in Guatemalan Sugarcane Workers
Source: J Occup Environ Med. 2018 Dec 20;61(3):239–50. doi: 10.1097/JOM.0000000000001529 (PMC6416034; doi:10.1097/JOM.0000000000001529)
Supplement: Supplemental Digital Content [file joem-61-239-s001.docx]

Supplement:

*Laboratory Methods*

Urine samples were collected in 120 mL sterile Nipro polypropylene containers and given to the research team in the field. Specific gravity was measured in the field using a digital refractometer (ATAGO PAL-10S digital refractometer, Tokyo, Japan). Immediately following analysis, samples were placed on ice and transported to the clinic. Within 2 hours, urine dipstick analysis was performed (Mission urinalysis reagent strips, ACON laboratories, San Diego, California USA) and semi-qualitative results for ketones, protein, nitrites, glucose, pH, leukocyte esterase and specific gravity were read and recorded by two trained personnel in accordance with product instructions. Immediately following dipstick analysis, the urine was aliquoted into Fisherbrand sterile polypropylene tubes without preservatives and frozen onsite to -20°C. At the end of each sample collection week, all urine samples were transported on ice to the Herrera-Llerandi laboratory. Urine samples were analyzed within 12 hours of reception. Creatinine was measured via kinetic alkaline picrate and uric acid via uricase with peroxidase and ascorbate oxidase (Abbott, Architect CI4100). Sodium, Potassium and Chloride were measured with direct ISE (I-Sens, I-Smart 30 Pro). Albumin was measured with florescence immunoassay (Boditech, I-Chroma). Magnesium and phosphorus were measured using standard laboratory technique (Roche Cobas Integra 400 Plus). Remaining aliquots were stored at -20°C and shipped to University of Colorado for additional analysis.

Post-shift blood samples were collected from each participant by a trained phlebotomist using one 4mL Vacuette® K3EDTA tube, followed by two 8 mL red top Vacuette® tubes. Following collection, all three tubes were gently inverted 10 times to ensure mixing of blood with the anticoagulant or serum clot activator. The blood samples were immediately placed on ice and transported within one hour to the clinic where they were centrifuged and stored at -20°C. At the end of each week, all blood samples were transported on ice to the Herrera-Llerandi laboratory. Upon arrival at the laboratory, samples were analyzed within 12 hours. Serum was analyzed for BUN, Creatinine, ALT, AST, and Uric acid by automated standard techniques (Abbot, Architect CI4100). Lactic acid was measured via ISE (Instrumentation laboratories, Gem Premier 3000). Hemoglobin A1c was determined with ionic exchange high pressure liquid chromatography (Biorad, D-10). Sodium and potassium were determined by standard automated techniques (I-Sens, I-Smart 30 Pro). Creatinine kinase was determined with CK-NAC serum start (DGKC) (Toche Cobas Integra 400 Plus).
